# A case of scalp avulsion with prolonged ischemic time: indocyanine green angiography can aid in predicting replant survival

**DOI:** 10.1186/s41038-019-0171-2

**Published:** 2019-12-09

**Authors:** Xin Huang, Zhichao Wang, Caiyue Liu, Shuchen Gu, Yashan Gao, Xiangwen Xu, Tao Zan

**Affiliations:** 1 Department of Plastic and Reconstructive Surgery, Shanghai Ninth People’s Hospital, Shanghai JiaoTong University School of Medicine, 639 Zhizaoju Road, Shanghai, 200011 People’s Republic of China; 2 Plastic and Reconstructive Surgery, Changzheng Hospital, Navy Military Medical University, Shanghai, People’s Republic of China

**Keywords:** Scalp avulsion, Tissue perfusion, Indocyanine green angiography, Microsurgical replantation, ICGA

## Abstract

**Background:**

Microsurgical replantation has become the most favorable treatment option for scalp avulsion. However, the accurate prediction of postoperative replant viability remains challenging.

**Case presentation:**

In this article, we showed that (indocyanine green angiography, ICGA) can provide a much more precise prediction of replant necrosis than conventional clinical assessment in a rare case of complete scalp avulsion with prolonged ischemia time.

**Conclusion:**

Clinical assessment of replant survival may be misleading in cases of complex tissue injuries and prolonged ischemic stress. This case provides insight into the promising utility of ICGA as an important adjuvant tool to better assess tissue perfusion and viability in scalp avulsion and possibly other types of replantation.

## Background

Scalp avulsions are rare case and are challenging to all reconstructive surgeons. Since the first successful scalp replantation performed in 1976 by Miller et al., microsurgical replantation has become the most favorable treatment option [1]. A successful replantation can well restore the hair-bearing aesthetic unit that is irreparable by other types of reconstruction [2], but replant failure may result in a suboptimal appearance, refractory wounds, and susceptibility to infections requiring secondary salvage surgery with prolonged hospitalization. How can the postoperative replant viability be precisely predicted? This problem still puzzles even the most experienced reconstructive surgeons.

Recently, indocyanine green angiography (ICGA) has been used for the real-time assessment of soft-tissue vascularity and perfusion after the intravenous injection of indocyanine green (ICG), which emits fluorescence when excited by a laser of a specific wavelength. In this article, for the first time, we showed that ICGA can provide a more precise prediction of replant survival than conventional clinical assessments in an intractable case of complete scalp avulsion with prolonged warm ischemia time.

## Case presentation

**Fig. 1:**
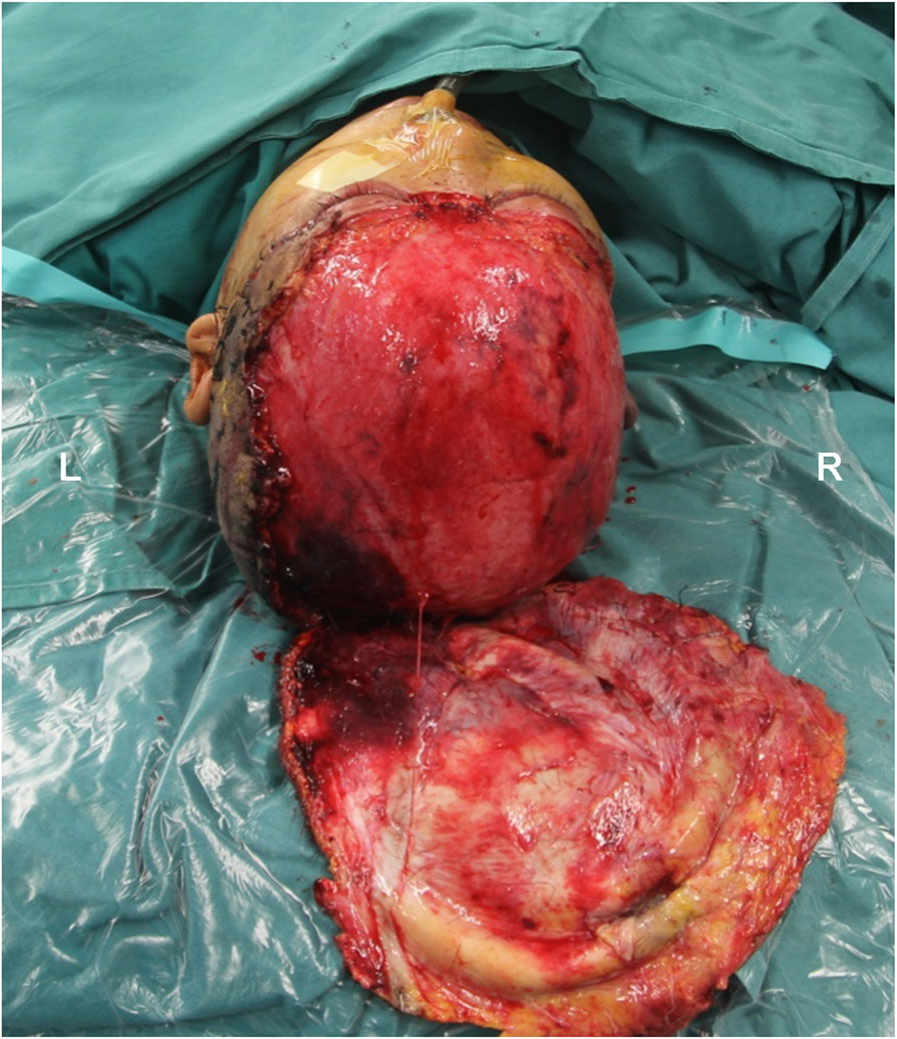
A preoperative photograph revealed total hairy scalp and forehead avulsion with only a connection in the nuchal area

A 42-year-old woman presented to our clinic 28 h after complete avulsion of her scalp by a rotating machine. Her scalp had been simply sutured in situ without anastomosis in a local hospital within 3 h after injury. After another 16 h of monitoring, there were no signs of improved perfusion in the scalp, so the patient was transferred to our hospital for salvage surgery. The patient was clearly conscious with stable vital signs. On physical examination, the avulsed scalp involved almost the entire hairy scalp and forehead, sparing the occipital region in the nuchal area (Fig. 1). An ophthalmic examination revealed normal eye movements and pupil light reflexes. The laboratory tests showed an elevated white blood cell count (15.6 × 10^9^/L, normal range 3.5–9.5 × 10^9^/L) and absolute neutrophil count (11.7 × 10^9^/L, normal range 1.8–6.3 × 10^9^/L), indicating stress and acute inflammatory status. The patient also had mild anemia (90 g/L).

**Fig. 2:**
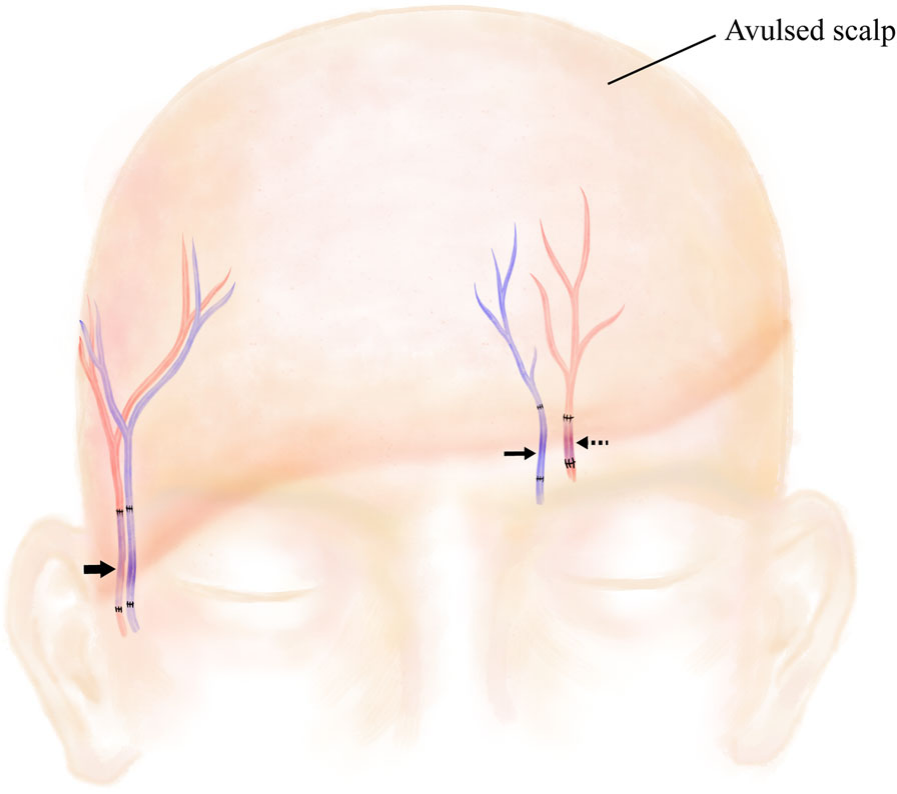
Schematic diagram of the microsurgical anastomosis of vascular stumps with vein grafts. (*Bold arrow*) The right temporal artery and vein; (*arrow*) the left supratrochlear vein; (*dashed arrow*) the left supraorbital artery

Measures were performed immediately to closely monitor the patient’s condition and prevent hemorrhagic shock. Considering the specificity of scalp tissue, immediate replantation was attempted despite the long ischemia time. After the onset of anesthesia, the patient was maintained in the supine position. The amputated flap was shaved and cleaned. The right temporal vessels, left supratrochlear vein, and left supraorbital artery were identified and carefully dissected out of the recipient area. Then, corresponding vascular stumps were identified and trimmed in the amputee. End-to-end anastomosis was performed between vessels in the recipient area and counterparts area with vein grafts from the forearm (Fig. 2). The total operative time for anastomosis was 7 h, limiting the overall warm ischemia time to 35 h.

**Fig. 3:**
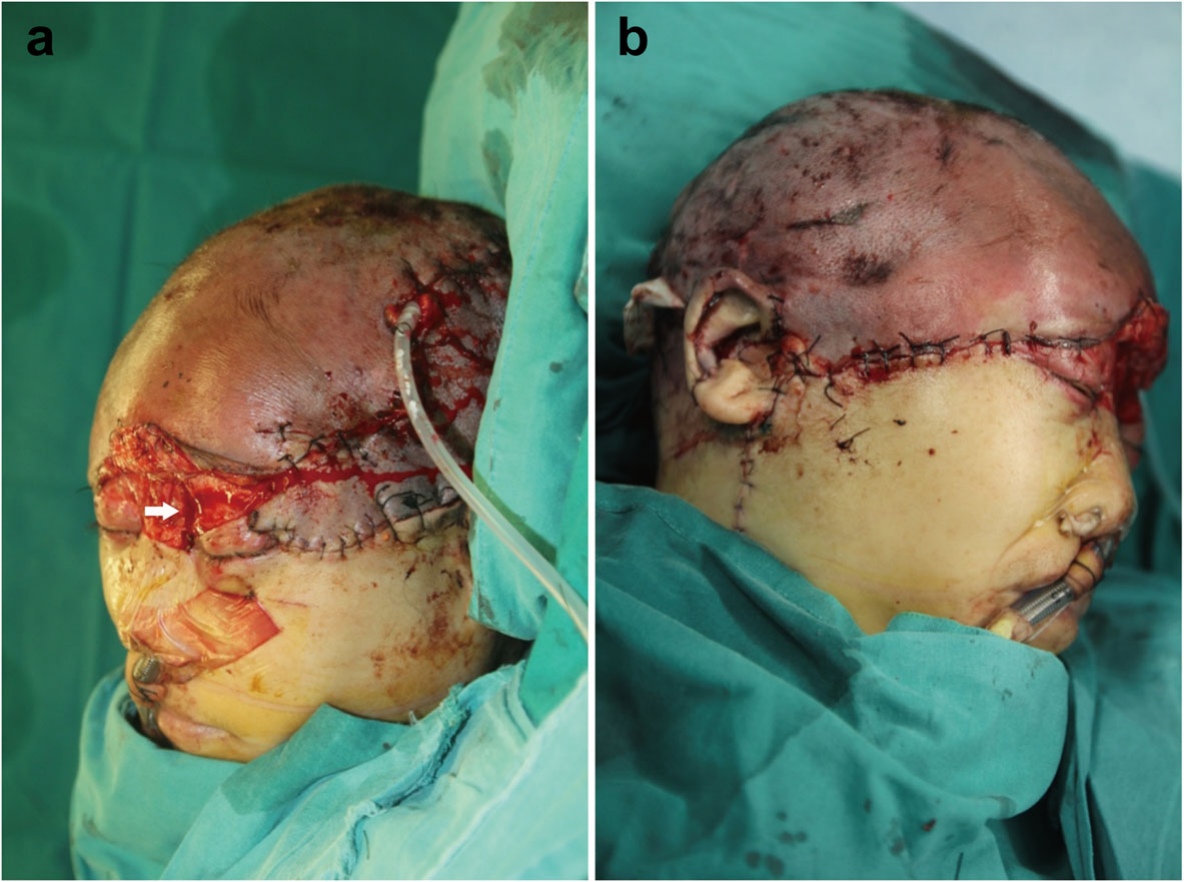
Intraoperative images after scalp replantation. After microsurgical anastomosis, **a** the left and **b** the right scalp tissue showed well perfusion according to clinical assessment. (*White arrow*) Please note the patency of the supraorbital artery and supratrochlear vein after anastomosis

**Fig. 4:**
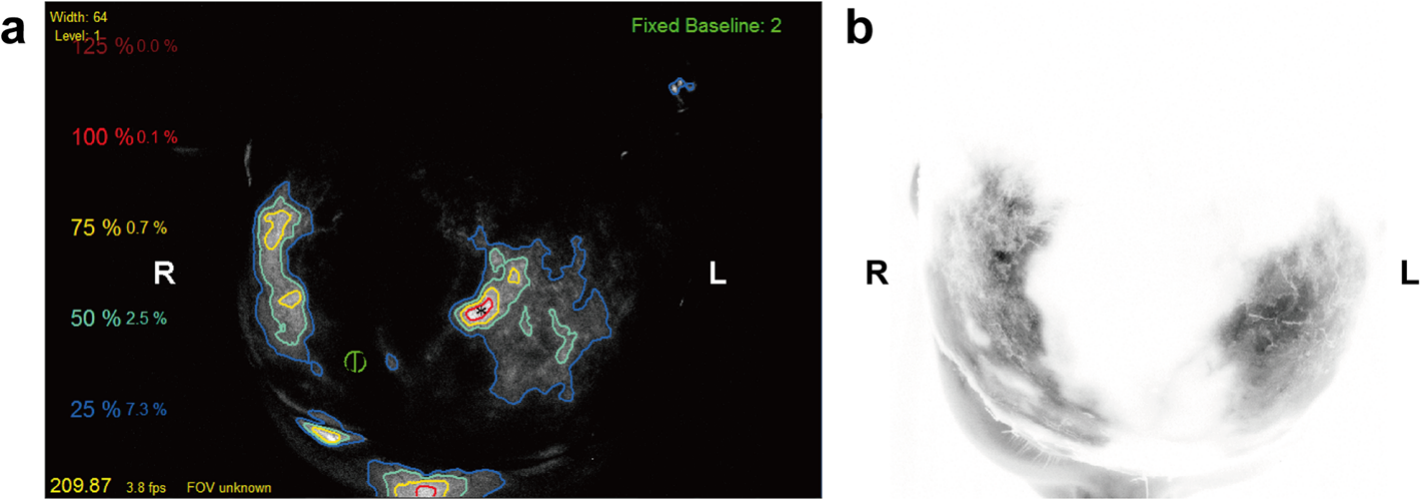
The intraoperative and postoperative indocyanine green angiography (ICGA) mapping results. **a** Intraoperative ICGA mapping with SPY-Q analysis of perfusion in the replanted scalp. Asterisk represents the left temporal area that is automatically selected as a reference with a 100% perfusion value. Areas with 25% perfusion relative to the maximal perfusion reference were traced with blue lines. Areas confined by the blue lines represent well-perfused tissue with more than 25% of the reference area perfusion. **b** The color reversal version of ICGA image when the necrotic area became mature. Please note the hypoperfused frontoparietal area represented by the color white

**Fig. 5:**
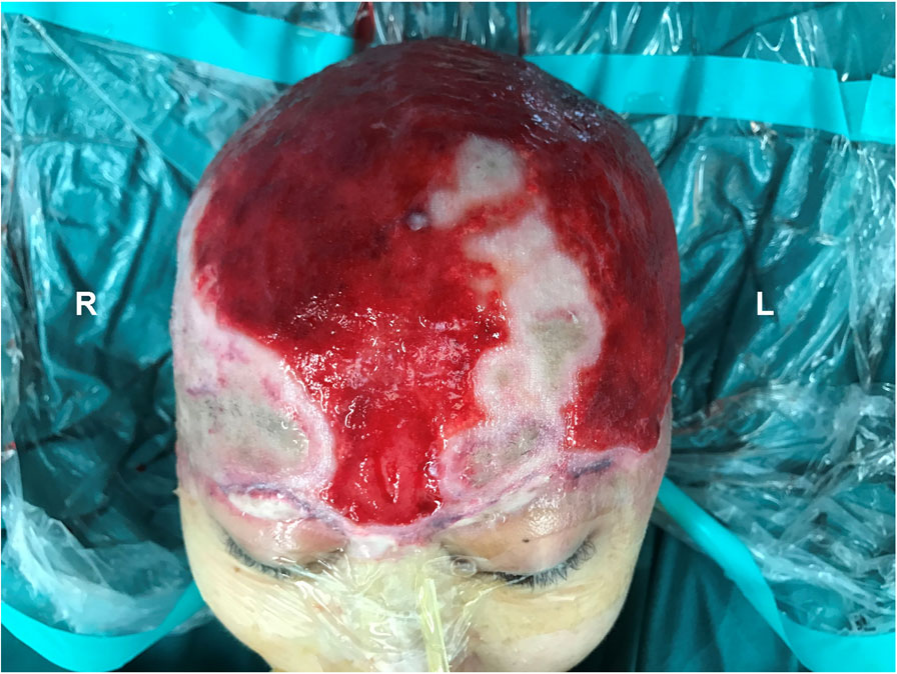
Intraoperative view after the debridement of necrotic tissues

**Fig. 6:**
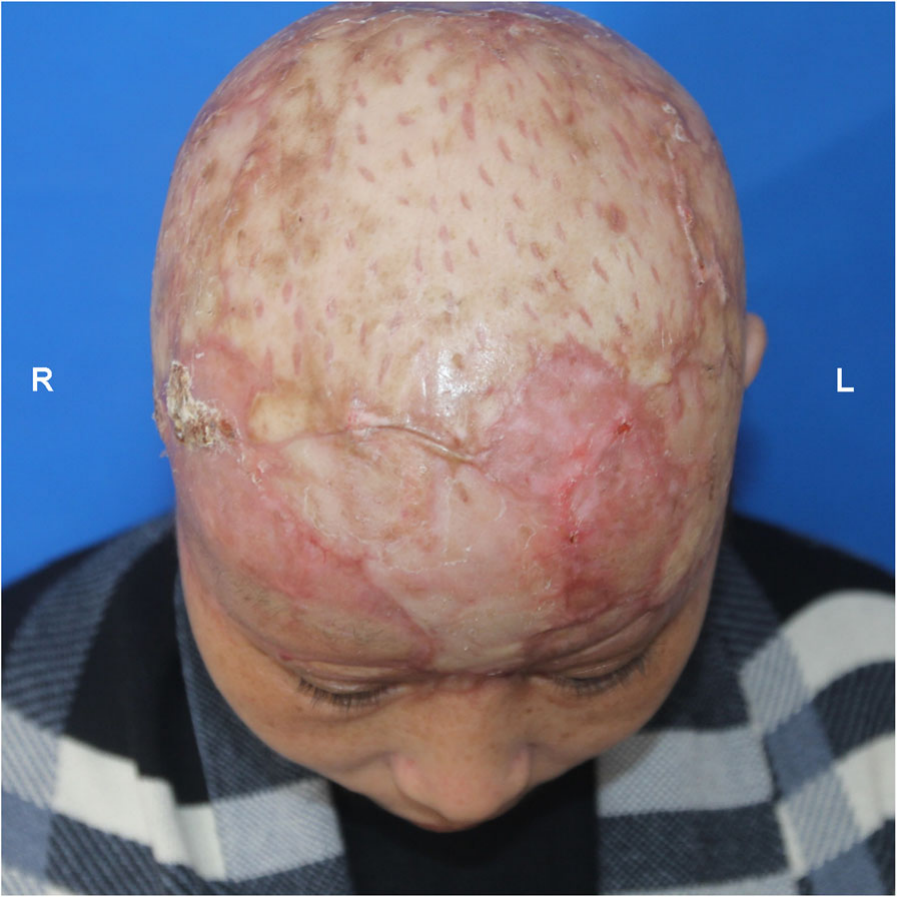
All skin grafts survived with acceptable cosmetic results at the 1-month follow-up

The entire scalp appeared well perfused, was flush red in color, and had positive pinprick bleeding and restored capillary refill (Fig. 3a, b). To confirm tissue perfusion, ICGA was conducted according to a previous study [3]. Briefly, a 2-mL ICG bolus (2.5 mg/mL, Dandong Yichuang Pharmaceutical Co., China) was injected through a peripheral intravenous line. The fluorescent detector (SPY imaging system, Novadaq Technologies Inc., Canada) was placed approximately 20 cm above the skin surface to acquire video data. Unexpectedly, ICGA mapping showed hypoperfusion in multiple areas that adequately perfused by the clinical assessment (Fig. 4a). We tried to find other suitable vessels for anastomosis, but the attempts were in vain. The patient was carefully monitored in the intensive care unit. Within the first week after the operation, the replanted scalp was swollen with considerable subcutaneous drainage. However, Doppler ultrasound revealed patent anastomosed vessels, and the flap was viable based on pinprick bleeding; thus, early postoperative ICGA was not planned. Unfortunately, partial necrosis occurred 1 week later and became matured in 1 week. Tissue perfusion was assessed again with ICGA mapping, which showed a wide range of hypoperfused frontoparietal areas with a pattern in accordance with the intraoperative ICGA mapping results (Fig. 4b). The patient underwent major debridement surgery, during which we confirmed that the necrotic area correlated accurately with the hypoperfused areas that had less than 25% of maximal perfusion revealed by ICGA mapping at the time of replantation using quantitative SPY-Q analysis (Fig. 4a and Fig. 5). Split skin grafting was performed with grafts from the back. All grafts survived, and the patient recovered uneventfully. Acceptable cosmetic results were achieved at the 1-month follow-up (Fig. 6). The patient was satisfied with her final appearance.

## Discussion

This emergent case of complete scalp avulsion featured a total of 35-h warm ischemia time, which to the best of our knowledge, is the longest ever reported in the literature [4, 5]. For the first time, we applied ICGA intraoperatively to assess the perfusion of the replanted scalp and showed the effectiveness of this method in predicting replant survival.

Currently, microvascular replantation is not technically challenging. However, predicting whether the replanted scalp can survive afterwards is still a point of confusion for reconstructive surgeons. The survival of replants is influenced not only by tissue perfusion after revascularization, but also by warm ischemia time [2]. Prolonged warm ischemia will make the replant susceptible to severe ischemia-induced necrosis and ischemia-reperfusion injury (IRI) [6, 7]. In the case of avulsion injuries, the damage to the inner vasculature of the tissue by mechanical force can further complicate the prediction of replant survival.

Clinical judgment remains the most commonly used standard for determining replant perfusion [8]. Therefore, the appearance of fresh red blood on pinprick testing is a strong indicator of sound perfusion, and flush blue blood after needle withdrawal suggests congestion requiring re-exploration. However, clinical assessments might be delaying or misleading in cases of complex tissue injuries and prolonged ischemic stress, as demonstrated by a major discrepancy between the clinical evaluation results and replant viability in this case [8]. Various technologies have been studied for a more objective evaluation of perfusion, such as laser Doppler imaging, fluorescein angiography, and thermography. However, these methods are seldomly used as emergency or intraoperative routine for various reasons ranging from complexity to insufficient sensitivity and/or specificity [8]. In contrast, ICGA is simple and reproducible and allows for the real-time observation of soft-tissue vascularity and perfusion. Several studies have validated the role of ICGA in the evaluation of skin flap viability [8–11]. ICGA is scarcely used for replantation [12]. Mothes et al. reported that ICGA more promptly detected perfusion deterioration than clinical evaluations and that ICGA was more relevant to the patients’ prognoses in 14 cases of hand and arm replantation than clinical evaluations [12].

In our study, the relative perfusion unit was generated with computer designated, best-perfused left temporal tissue as a reference (Fig. 4a). By superimposing the ICGA mapping results on the clinical image, we could determine that the contoured areas with less than 25% of maximal perfusion were prone to necrosis, which is similar to the results described in a previous study [9]. Because suitable vascular stumps for anastomosis were unavailable, we did not perform any additional anastomosis.

In this study, we showed that ICGA mapping is a more sensitive method for detecting tissue perfusion and provides a more accurate prediction of replant survival than conventional clinical assessments. However, this study reports a single case with limited representativeness. This hypothesis could be further confirmed by case series studies or controlled trials, and ICGA may be instrumental to the management of patients with scalp avulsion. On the one hand, timely tissue reduction or skin grafting with autografts from the avulsed scalp could be performed on poorly perfused areas to avoid postoperative replant failure [13]. On the other hand, postoperative ICGA could sensitively report compromised perfusion so that immediate interventions could be applied to rescue the necrotizing tissues. Additionally, ICGA is more convenient and repeatable than other methods, making ICGA suitable for emergency and intraoperative scenarios. Unfortunately, the ICG imaging device is quite expensive with limited popularization right now. In addition, the results should be interpreted with caution and should be validated with different imaging platforms. Finally, the modification of the threshold to achieve a more precise prediction is warranted.

## Conclusions

Our experience provides insight into the promising utility of ICGA as an important adjunct tool for evaluating tissue perfusion in scalp avulsion and possibly other types of replantation to guide clinical decision making, shorten hospitalization time, and improve clinical outcome and patient satisfaction.

## Data Availability

The patient and all list authors agreed to share the data included in this case report.

## Abbreviations

ICGA: Indocyanine green angiography
IRI: Ischemia-reperfusion injury
